# Effects of occupational therapy synchronized with dual transcranial direct current stimulation on upper limb function and electroencephalography power in subacute stroke patients: A randomized, double-blind, controlled study

**DOI:** 10.1371/journal.pone.0320142

**Published:** 2025-03-18

**Authors:** Ling Gao, Fengming Chu, Xuan Liu, Jie Chen, Ming Zhang, Yuming Zhang

**Affiliations:** 1 Department of Rehabilitation Medicine, The Affiliated Xuzhou Rehabilitation Hospital of Xuzhou Medical University, Xuzhou, China; 2 Department of Rehabilitation Medicine, Xuzhou Central Hospital/Xuzhou Clinical College, Xuzhou Medical University, Xuzhou, China; University of Zanjan, IRAN, ISLAMIC REPUBLIC OF

## Abstract

**Background:**

Occupational therapy (OT) and transcranial direct current stimulation (tDCS) are both important methods for promoting the recovery after stroke. There are limited researches that simultaneously apply both methods and investigate their effects on upper limb function.

**Objective:**

To investigate the effects of OT synchronized with dual tDCS on upper limb motor function and Electroencephalogram (EEG) power in subacute stroke patients.

**Methods:**

Forty-five subacute stroke patients were randomly assigned to control group (n = 23) and experimental group (n = 22), receiving sham and real dual tDCS concurrent with OT respectively, five times a week, for a duration of two weeks. Upper limb motor function and cortical EEG power were evaluated by Fugl-Meyer Assessment Upper Extremity (FMA-UE), Modified Barthel Index (MBI) and Action Research Arm Test (ARAT), Delta/Alpha Ratio (DAR) and pairwise derived Brain Symmetry Index (pdBSI) at baseline and two weeks.

**Results:**

Finally, a total of 39 patients completed the study and were included in the analysis. The results revealed that participants in the experimental group showed a significant better evolution for FMA-UE (p < 0.001), MBI (p = 0.034), DAR in the primary motor cortex (M1) area (p = 0.022) and pdBSI (p = 0.025) compared to the control group.

**Conclusions:**

In subacute stroke patients, the central-peripheral combined stimulation approach, which involves dual tDCS (central stimulation) and synchronous OT (peripheral sensory-motor stimulation) enhanced the effects of OT alone, leading to greater improvements in upper limb function and normalization of brain activity.

**Trial registration:**

This trial was registered in the Chinese Clinical Trial Registry (No. ChiCTR2400082749).

## Introduction

Stroke is a chronic non-communicable disease and has become the second leading cause of disability and death among adults worldwide [1]. In China, due to the aggravation of population aging and the influence of lifestyle, risk factors, etc., the burden of stroke is increasing year by year [2]. After a stroke, patients often suffer from various functional disorders, such as motor disorders, speech disorders, swallowing disorders, etc. Approximately 75% of survivors have upper limb motor dysfunction [3]. The treatment of upper limb motor dysfunction has a long cycle, poor prognosis, and is more difficult to recover compared to lower limb impairments. Only 5–20% of patients are able to achieve complete recovery [4], which severely limits their functional activities and social participation, leading to a decreased quality of life. Existing treatment options for upper limb function and activities of daily living (ADL) after stroke have not shown significant therapeutic effects, and patients often lack motivation and cooperation. Therefore, there is an urgent need to develop and test new and effective upper limb training protocols.

Non-invasive brain stimulation techniques (NIBS), such as transcranial direct current stimulation (tDCS) and transcranial magnetic stimulation (TMS), are considered one of the most promising new technologies in stroke rehabilitation [5]. TMS generates a fluctuating magnetic field through an insulated coil placed on the scalp, delivering short and intense electrical pulses to the brain tissue to modulate the excitability of neurons in the stimulated area. tDCS delivers a constant weak direct current (1–2 mA) to the cerebral cortex through two electrodes placed on the scalp, regulating the activity of neurons in the cerebral cortex, changing the resting membrane potential, and causing focal reversible polarization of brain regions, thereby enhancing the brain plasticity of stroke patient [6,7]. Compared with TMS, tDCS offers advantages such as affordability, accessibility, safety, portability, and mild side effects [8], making it a viable choice for future research. The therapeutic effect of tDCS depends on multiple factors, and one important factor is polarity[9]. During anodal stimulation, the current flows inward from the anode into the cortex, causing depolarization of nerve cells and thus increasing the excitability of the affected side cortex. During cathodal stimulation, the current flows outward from the cathode out of the cortex, causing hyperpolarization of nerve cells and thus reducing the excitability of the healthy side cortex [10]. Bipolar stimulation acts on both cerebral hemispheres simultaneously, causing excitability changes in the cortices of both sides [11]. A large number of studies have confirmed the role of tDCS in the recovery of upper limb motor disorders after a stroke. In the study by Unger et al. [12], compared with the sham operation group, after applying anode tDCS to the ipsilateral premotor cortex, the interhemispheric functional connectivity of patients was enhanced, which was related to the upper limb motor recovery of patients with moderate to severe chronic stroke. These changes were confirmed by functional magnetic resonance imaging. In the study by Bolognini et al. [13], compared with the sham stimulation group, patients showed greater improvement in upper limb muscle strength after receiving dual tDCS treatment.

Occupational therapy (OT) is an important part of stroke patient rehabilitation. It aims to improve limb function, maintain or restore the highest possible level of independence, enhance the ability of ADL, and facilitate patients’ return to family, society, and work through targeted, purposeful, and personalized activities [14,15]. Usually, the execution of these purposeful activities is both the overall goal and the basis of the intervention [16]. A study in Spain investigated 60 adults who returned home after a stroke and found that OT could effectively improve the quality of life, enhance perceptual and cognitive skills, enhance independence, and reduce the level of depression of stroke patients. It can be regarded as an applicable non-drug treatment tool, leading to positive outcomes for patients after a stroke [17].

In 2016, Chinese scholar Jia Jie proposed a new rehabilitation concept: “central-peripheral-central” closed-loop intervention [18]. Its core concept is the overall planning of central intervention and peripheral intervention, complementing the functions of “peripheral intervention” and “central intervention”. Based on the plasticity of the brain and the remodeling of neural pathways, it promotes central remodeling and peripheral control, and then promotes functional recovery. A number of studies have confirmed that the combination of central intervention and peripheral intervention can have a beneficial impact on the recovery of upper limb motor function and ability of ADL in stroke patients [19,20]. Then, if tDCS is used as a central intervention and OT as a peripheral intervention, and the two are combined and applied to stroke patients simultaneously, will it also produce good results? The result is unknown because, as far as the author knows, this has not been fully investigated to a large extent. Only two studies have conducted similar explorations. Therefore, it is necessary to further explore the effectiveness of this synchronous combination of central and peripheral interventions on upper limb recovery, use different measurement indicators to evaluate a more comprehensive range of activities, examine the activity of the cerebral cortex to help elucidate the underlying mechanisms of neural modulation and provide meaningful insights. We hypothesized that synchronous dual tDCS in combination with OT can enhance the benefits of OT, enhance the brain plasticity of subacute stroke patients to a greater extent, promote upper limb function recovery, and facilitate the normalization of brain electrical power. To validate our hypothesis, we utilized cost-effective and minimally invasive electroencephalography (EEG) to record cortical activity, as it is highly sensitive in detecting typical brain rhythm abnormalities in stroke [21].

## Methods

### Participants

**Fig 1:**
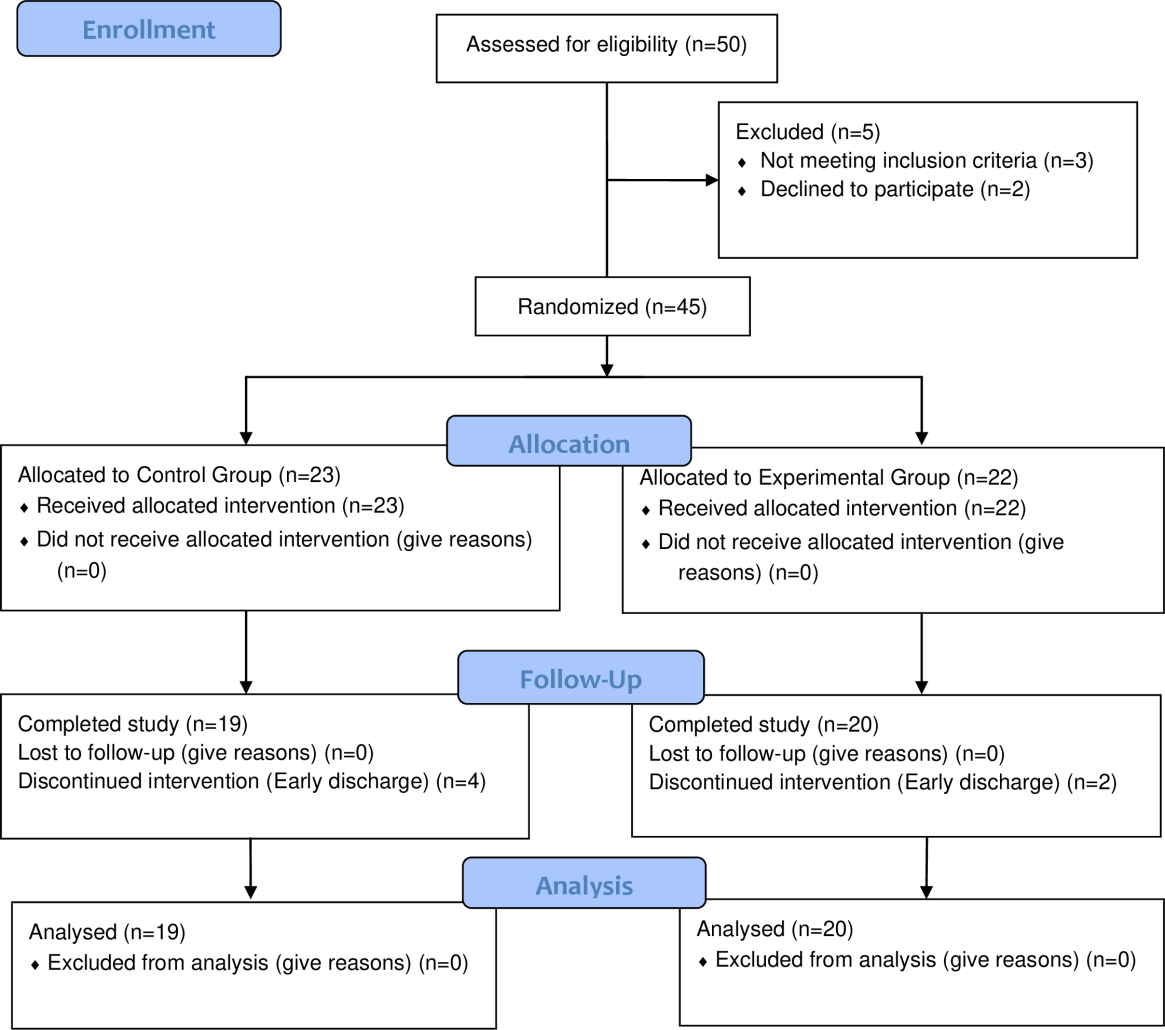
Consolidated standards of reporting trials flow chart.

The authors confirm that all ongoing and related trials for this intervention are registered. This study was a randomized controlled trial, approved by the Ethics Committee of The Affiliated Xuzhou Rehabilitation Hospital of Xuzhou Medical University (NO.XK-LSW-20240321-018), and registered in the Chinese Clinical Trial Registry (www.chictr.org.cn) under the registration number ChiCTR2400082749. From March 22, 2024 to July 28, 2024, the individuals were recruited, evaluated, and completed treatment from The Affiliated Xuzhou Rehabilitation Hospital of Xuzhou Medical University and Xuzhou Central Hospital. All patients provided written informed consent prior to enrollment. A CONSORT diagram is illustrated in Fig 1.

Participants who met the following criteria were included in the study: (a) diagnosis with first unilateral subcortical stroke and has been confirmed by a head CT or MRI scan; (b) age between 25 and 80 years; (c) stroke with a stable clinical course lasting between 7 days and 6 months; (d) Brunnstrom staging of the upper limb and hand ≤  Stage IV; (e) Mini-Mental State Examination (MMSE) score ≥  21, indicating clear consciousness and ability to cooperate with the necessary examinations and treatment.

Participants who met the following criteria were excluded from the study: (a) progressive stroke or subarachnoid hemorrhage with unstable condition; (b) patients with severe cardiovascular diseases, primary hypertension, internal medicine diseases, mental trauma, or cognitive impairments; (c) patients with metallic implants in the intracranial or treatment area, or with skull defects; (d) patients with epilepsy or brain tumors; (e) patients with poor compliance or unwillingness to cooperate with rehabilitation training.

A total of forty-five subacute stroke patients of both sexes met the criteria and participated in this research. Group allocation was determined by a computer-generated random number sequence. The random numbers were placed in opaque sealed envelopes with corresponding numbers, which were kept by an investigator involved in participant recruitment. Patients were numbered in order of enrollment, and envelopes with matching numbers were opened to assign the corresponding treatment groups. All patients were allocated to either the control group or the experimental group in the same proportion. The control group (n = 23) received sham dual tDCS concurrent with OT, while the experimental group (n = 22) received real dual tDCS concurrent with OT. The intervention was blind to participants, OT therapists, outcome assessor and statistician, with only the tDCS operator being unblinded.

### Outcome measures

All assessments were performed by a clinically trained therapist who was blinded to group assignments and did not participate in the intervention. The baseline data, which refers to the pre-treatment data, was collected at the same time as the patient’s enrolment. After the treatment period concluded, which was two weeks later, post-treatment data was collected. The following clinical indicators and EEG data were all collected at two time points pre- and post-treatment.

#### Clinical evaluation.

The primary outcome measure was Fugl-Meyer Assessment Upper Extremity (FMA-UE). FMA-UE is considered the gold standard for assessing motor impairment or motor control [22]. It includes 33 items that evaluate reflexes, coordination, and isolated movements of the upper limb, with a total score of 66. A higher score indicates better upper limb function [23]. The secondary outcome measures were Modified Barthel Index (MBI) and Action Research Arm Test (ARAT). MBI includes 10 items related to basic ADL, such as dressing, eating, and bathing. The total score is 100, and a higher score indicates better self-care ability for the patient [24]. ARAT consists of 19 items that assess the ability to grasp, grip, pinch, and perform gross movements of the upper limb. The total score is 57, with a higher score indicating better upper limb function [25].

#### EEG data acquisition and analysis.

**Fig 2:**
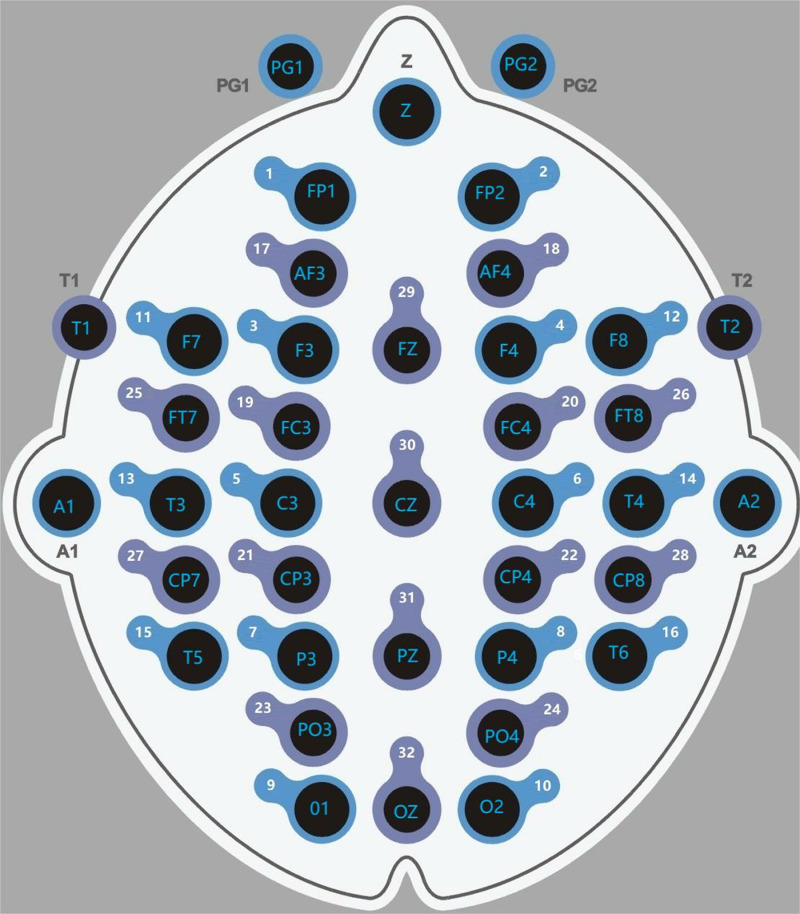
Schematic diagram of electrode placement for the EEG cap.

The JY-2440 Digital Electroencephalography Topographic Mapping Instrument (Jiangsu Jinyuan Medical Technology Co., Ltd., China) was used to collect the patient’s resting-state EEG data with closed eyes for a minimum of 5 minutes. As shown in Fig 2, participants wore an EEG cap with 32 active electrodes positioned at FP1, FP2, F3, F4, C3, C4, P3, P4, O1, O2, F7, F8, T3, T4, T5, T6, AF3, AF4, FC3, FC4, CP3, CP4, PO3, PO4, FT7, FT8, CP7, CP8, FZ, CZ, PZ, and OZ, according to the international 10/20 system. Reference electrodes were placed at the bilateral earlobe locations, and a ground electrode was placed at the Z location. The EEG signals were filtered within the range of 0–30 Hz, with a time constant of 0.03 s and a sampling rate of 30 mm/s.

Data analysis was performed using MATLAB R2015a software and the EEGLAB toolbox. Independent Component Analysis (ICA) algorithm was used to correct artifacts. Fast Fourier Transform (FFT) was applied to convert the EEG signals into average power spectra for each electrode. The power spectra were then summed within the frequency bands of 1–4 Hz, 4–8 Hz, 8–12 Hz, and 13–30 Hz to obtain the average power values for delta, theta, alpha, and beta bands, respectively. These power spectra were used to calculate the following two quantitative indices.

(1) Delta/Alpha Ratio (DAR) [26]: It is defined as the ratio between the absolute power of the delta band and the alpha band in EEG analysis.

$$DAR=\frac{\delta }{\alpha }$$

The “Average Scalp Power Spectra” was calculated by averaging the power across all scalp electrodes for each frequency iteration. The global DAR was then determined as the ratio between the average power of the delta band and the alpha band in the scalp power spectra.Similarly, the DAR in the primary motor cortex (M1) area (we refer to it as M1-DAR) was calculated using the average power from three electrodes: C3, C4, and CZ. Finally, at the group level, topographic maps of the global DAR and M1-DAR were generated to visualize the data, show the spatial distribution of these ratios across the scalp and provide detailed representations of DAR values across different electrode locations, aiding in the localization of specific active brain regions.

(2) pairwise derived Brain Symmetry Index (pdBSI) [27]: It is defined as the average absolute normalized difference in spectral power between homologous channel pairs of the left and right hemispheres. It represents the average absolute value of the difference in power between the two hemispheres within the frequency range of 1–25 Hz, and reflects the symmetry between the two hemispheres of the brain.

$$pdBSI=\frac{1}{NM}\overset{M}{\underset{j=1}{\sum }}\overset{N}{\underset{i=1}{\sum }}|\frac{R_{ij}-L_{ij}}{R_{ij}+L_{ij}}|$$

Here, $R_{ij}$ and $L_{ij}$ respectively represents the power of right and left channel from the homologous channel pair (for channel pair j =  1, 2,..., M) at frequency i =  1, 2,..., N. The pdBSI ranges from 0 to 1, where a pdBSI value of 0 indicates complete symmetry (total symmetry), and a value of 1 represents complete asymmetry (maximum asymmetry).

### Intervention

All the patients were given OT treatment by OT therapists who were blinded to the group assignments. Before commencing rehabilitation, patient goals and preferences were determined through communication with the patients and their families. Based on the individual backgrounds, symptoms and specific conditions, personalized training programs were designed, incorporating various activities such as joint movements of the upper limb (such as adduction and abduction of the shoulder joint, flexion and extension), upper limb muscle strength and endurance training (such as lifting a gymnastic rod and holding it), fine finger movement training (such as finger extension, gripping, opposition), and training for ADL (such as combing hair, buttoning clothes, eating), etc. Each session lasted for 40 minutes and was conducted once daily, five times a week, for a duration of two weeks. Additionally, the experimental group received real dual tDCS at the same time while the control group received a sham stimulation. tDCS was applied using the A620P portable tDCS device (Nanjing Wogao Medical Technology Co., Ltd., China). The patients were comfortably seated with their whole body relaxed. The placement of tDCS electrodes was determined according to the international 10/20 system for electrode placement. Bipolar stimulation was applied, with the anode placed over the ipsilateral M1 area and the cathode placed over the contralateral M1 area [28]. The stimulation electrodes used standard configuration 5 cm ×  5 cm saline-soaked sponge electrode pads, which were secured to the patient’s head using elastic straps. The stimulation intensity was set at 2.0 mA, each session lasted for 20 minutes and was conducted once daily, five times a week, for a duration of two weeks. The same stimulation setup was used for sham tDCS, but the stimulation stopped after 30 seconds.

### Statistical analysis

The study protocol was based on a previous study published by Nair et al. That study investigated the effect of concurrent use of cathodal tDCS during OT on promoting the recovery of upper limb function in stroke patients [29]. A total of 14 patients were recruited and randomly assigned to two groups, receiving real (cathodal) tDCS +  OT and sham tDCS +  OT respectively. The focus was on the changes in FMA-UE scores and joint range of motion. The results showed that the synchronous combination of cathodal tDCS and OT led to a significant improvement in post stroke motor function. The data of the above-mentioned study was used for power analysis. Taking the FMA-UE scores as the primary outcome, the sample size required for this study was calculated. The calculation was performed using PASS 15.0 software, assuming a power of 0.80 for a two-group comparison. The effect size (Cohen’s d) for FMA-UE was estimated to be 0.47. Considering an estimated dropout rate of 20%, the conclusion was that 44 participants were required.

All analyses were performed by an independent assessor using SPSS 27.0 software. The normality of all continuous variables was assessed using the Shapiro-Wilk test. The homogeneity of variances was tested using the Levene’s test. For baseline evaluations, Fisher’s exact test was used to compare categorical data, while the independent samples t-test and Mann-Whitney U test were used to compare continuous data. For continuous variables that met the criteria of normal distribution and homogeneity of variances, two-way mixed analyses of variance (ANOVA) was performed. We chose to use it because it could allow for a more comprehensive and effective analysis of the changes between the experimental and control groups at different time points, enabling us to assess the impact of both factors and their interaction on the results. Time was used as the within subject factor (before and after treatment), and group was used as the between subject factor (control group and experimental group). In the case of a significant time ×  group interaction effect, Bonferroni adjustment was applied to the post-hoc pairwise comparisons of time and group to determine the comparisons that led to the differences. For continuous variables that did not meet the normality assumption, the Wilcoxon signed-rank test was used for within-group comparisons, and the Mann-Whitney U test was used for between-group comparisons. A p-value of <  0.05 was considered statistically significant.

## Results

A feasibility assessment was conducted on 50 patients, of which 45 were underwent randomization. Finally, a total of 39 participants completed the designated intervention measures. At baseline, there were no significant differences observed between the two groups in terms of age, gender, time since stroke event, lesion side, or stroke subtype (Table 1). All participants tolerated the study well, and no significant adverse reactions were reported in either group. The actual training time was similar across all groups.

**Table 1:** Baseline characteristics between the two groups.

Characteristic	Control group	Experimental group	p-value
**Age (years)**			0.352
Mean (SD)	61.05(11.58)	57.00(14.95)	
**Sex**			1.000
Male	13(68.42%)	14(70.00%)	
Female	6(31.58%)	6(30.00%)	
**Time since stroke (days)**			0.607
Median (Q1, Q3)	46(23.00, 60.00)	40(24.50, 50.75)	
**Impairment side**			0.320
Left hemiplegia	14(73.68%)	11(55.00%)	
Right hemiplegia	5(26.32%)	9(45.00%)	
**Stroke type**			0.716
Ischaemic	15(78.95%)	14(70.00%)	
Haemorrhagic	4(21.05%)	6(30.00%)	

SD: standard deviation. Q1:1st quartile. Q3: 3rd quartile

### Clinical assessment

**Fig 3:**
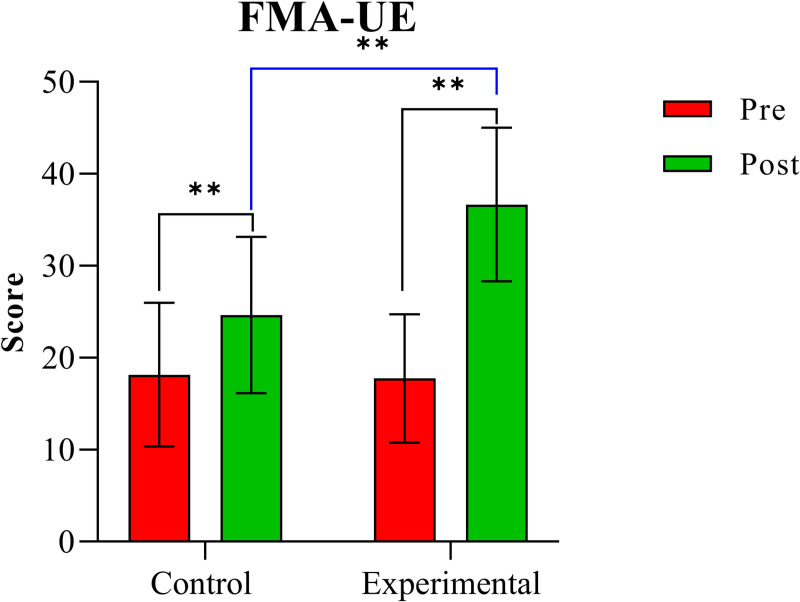
Comparison of FMA-UE scores between the two groups. *: p <  0.05, **: p <  0.001.

**Fig 4:**
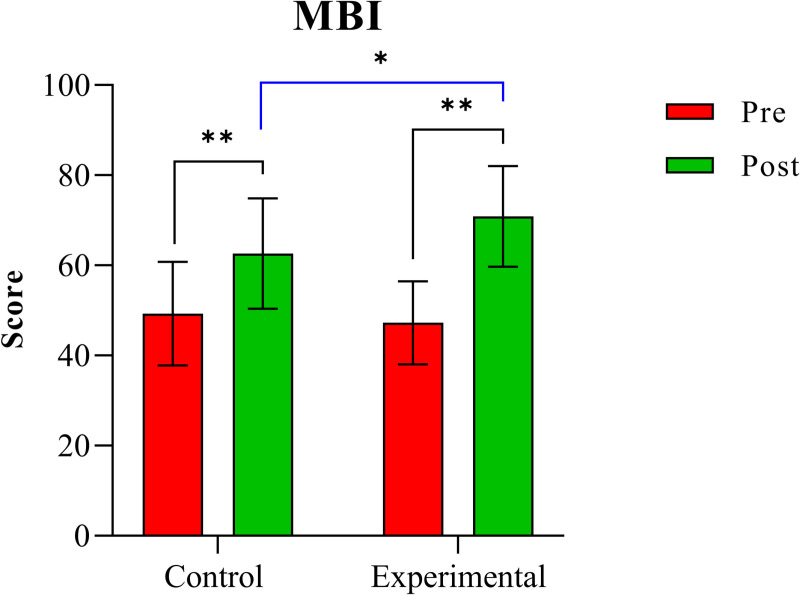
Comparison of MBI scores between the two groups. *: p <  0.05, **: p <  0.001.

Table 2 displays the results of the two-way mixed ANOVA for three clinical indicators to examine changes in scores from baseline to final evaluation. Significant interactions between time (within-subject factor) and group (between-subject factor) were found for two variables, with post-hoc test results indicating significant differences for these variables. The table includes mean and standard deviation values for initial and final assessments in each group, along with analysis results showing significant differences between the two groups, including p-values, F-values, and effect sizes. Furthermore, Figs 3 and 4 illustrate the changes in variables with significant interaction factors (time ×  group).

**Table 2:** Comparison of clinical evaluation outcomes between the two groups.

Variable/ Time	Control group	Experimental group	Time effect	Group effect	Time×Group effect	POST-HOC
	Mean ± SD	Mean ± SD				Exp vs Con
**FMA-UE**						
Pre-treatment	18.16 ± 7.82	17.75 ± 6.98	F = 195.402 p < 0.001^*^ ղ^2^ = 0.841	F = 5.567 p = 0.024^*^ ղ^2^ = 0.131	F = 42.326 p < 0.001^*^ ղ^2^ = 0.534	p = 0.864
Post-treatment	25.05 ± 8.55	36.65 ± 8.36				p < 0.001^*^
**MBI**						
Pre-treatment	49.26 ± 11.51	47.25 ± 9.18	F = 315.682 p < 0.001^*^ ղ^2^ = 0.895	F = 0.854 p = 0.361 ղ^2^ = 0.023	F = 24.352 p < 0.001^*^ ղ^2^ = 0.397	p = 0.549
Post-treatment	62.63 ± 12.22	70.90 ± 11.17				p = 0.034^*^
**ARAT**						
Pre-treatment	8.95 ± 5.71	10.20 ± 6.71	F = 210.974 p < 0.001^*^ ղ^2^ = 0.851	F = 1.190 p = 0.282 ղ^2^ = 0.031	F = 2.920 p = 0.096 ղ^2^ = 0.073	
Post-treatment	17.63 ± 8.13	21.20 ± 8.02				

SD: standard deviation

*Significant effect (P <  0.05).

The results of the two-way mixed ANOVA indicated a significant time ×  group interaction effect for FMA-UE (F =  42.326, η² =  0.534, p <  0.001). Post-hoc comparisons indicated significant increases in FMA-UE scores for both the control and experimental groups over time (both p <  0.001). There was no significant difference in FMA scores between the two groups before treatment (p =  0.864), but a significant difference was observed after treatment (p <  0.001).

For MBI, a significant time ×  group interaction effect was found (F =  24.352, η² =  0.397, p <  0.001). Post-hoc comparisons revealed significant increases in MBI scores for both the control and experimental groups over time (both p <  0.001). There was no significant difference in MBI scores between the two groups before treatment (p =  0.549), but a significant difference was observed after treatment (p =  0.034).

### EEG assessment

Table 3 presents the results of non-parametric tests for three EEG indicators. Two variables exhibited significant within-group differences. Before treatment, there were no significant intergroup differences in the three variables between the control and experimental groups. After treatment, two variables showed significant intergroup differences.

**Table 3:** Comparison of EEG evaluation outcomes between the two groups.

Variable/ Time	Control group	Experimental group	Within- Group (Con)	Within- Group (Exp)	Between groups
	Median (Q1, Q3)	Median (Q1, Q3)			
**global DAR**					
Pre-treatment	3.02 (2.04, 7.75)	3.89 (2.20, 5.06)	p = 0.136	p = 0.104	p = 0.857
Post-treatment	3.12 (1.68, 4.60)	2.68 (1.36, 4.09)			p = 0.708
**M1-DAR**					
Pre-treatment	3.12 (1.77, 6.07)	3.63 (2.04, 5.41)	p = 0.355	p = 0.019^*^	p = 0.667
Post-treatment	3.16 (1.84, 4.48)	1.85 (0.86, 2.62)			p = 0.022^*^
**pdBSI**					
Pre-treatment	0.28 (0.23, 0.40)	0.30 (0.21, 0.46)	p = 0.319	p = 0.006^*^	p = 0.665
Post-treatment	0.27 (0.20, 0.36)	0.19 (0.12, 0.26)			p = 0.025^*^

Q1:1st quartile; Q3: 3rd quartile.

*Significant effect (P <  0.05).

**Fig 5:**
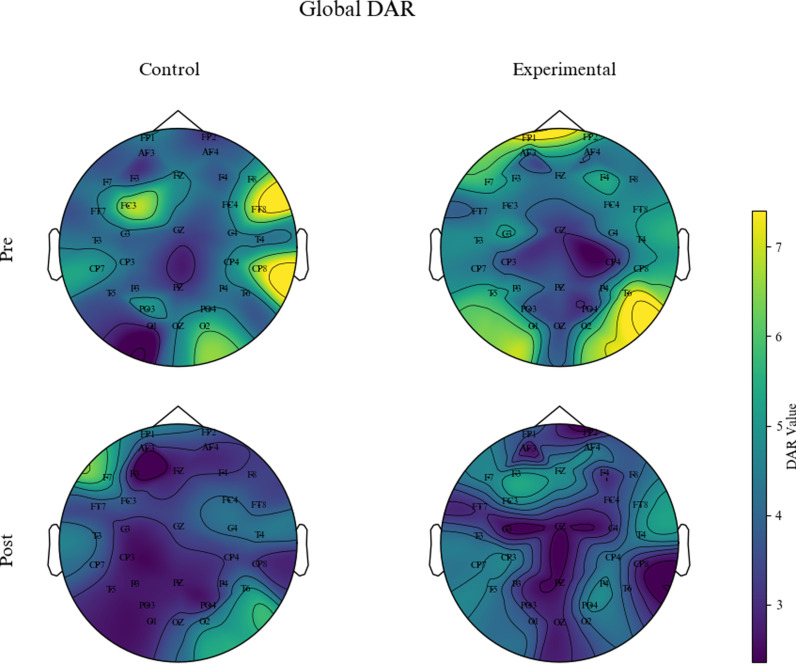
Topographical map of global DAR between the two patient groups.

**Fig 6:**
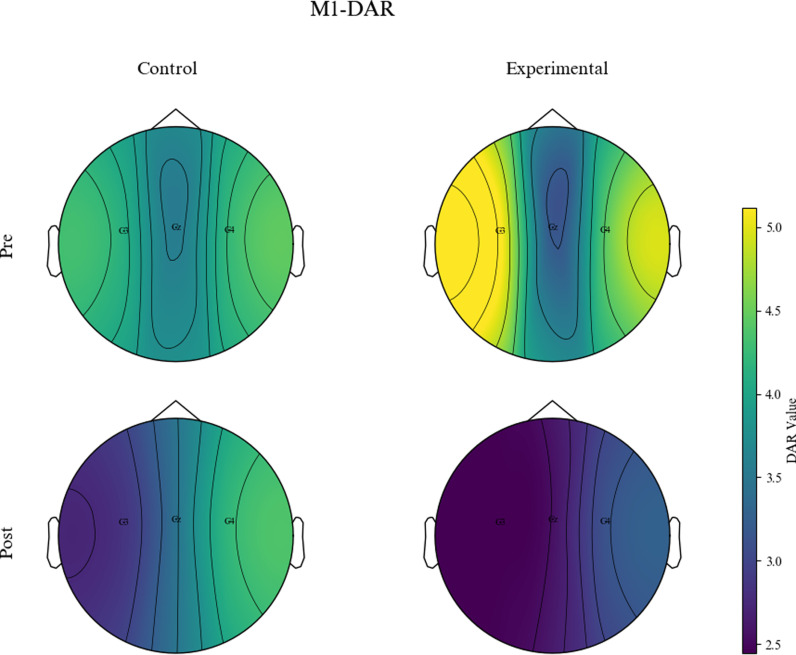
Topographical map of M1-DAR between the two patient groups.

Results of the Wilcoxon signed-rank test indicated a significant decrease in M1-DAR for the experimental group over time (p =  0.019). The Mann-Whitney U test showed no significant between-group difference in M1-DAR before treatment (p =  0.667). However, a significant between-group difference in M1-DAR was observed after treatment (p =  0.022). Figs 5 and 6 present topographical maps for global DAR and M1-DAR respectively, providing detailed insights into the spatial distribution of DAR values across different electrode locations on the scalp, aiding in the localization of specific active brain regions.

Over time, there was a significant decrease in pdBSI for the experimental group (p =  0.006). Before treatment, there was no significant difference in pdBSI between the control and experimental groups (p =  0.665). However, a significant between-group difference in pdBSI was observed after treatment (p =  0.025).

## Discussion

The results of this randomized, double-blind controlled trial demonstrated that the central-peripheral combined stimulation approach, which involves dual tDCS (central stimulation) applied to both hemispheres and synchronous OT (peripheral sensory-motor stimulation), enhanced upper limb function in subacute stroke patients and promoted the normalization of EEG power. The effectiveness of this approach surpassed a sham tDCS protocol with similar intensity concurrent with OT, indicating its efficacy in subacute stroke patients and supporting the research hypothesis.

### Clinical assessment

FMA-UE and MBI exhibited significant time ×  group interaction effects. Post-hoc comparisons indicated significant differences in FMA-UE and MBI between the two groups after treatment. Real dual tDCS combined with OT was the most effective intervention for improving upper limb motor performance. Several factors may account for these results: Firstly, recovery after stroke is slow, and there is substantial variability in the recovery of upper limb voluntary movement. This process is closely related to brain plasticity phenomena, including excitability changes in the neuronal circuits surrounding the lesion and the formation of new functional neuronal connections [30]. Neuroscientific evidence suggested that the introduction of a learning process, such as rehabilitation training, after stroke enhanced neural plasticity and had strong implications for compensating for functional deficits in the affected hemisphere and promoting functional recovery [31]. Unfortunately, stroke-related brain plasticity is transient, reaching its maximum during the subacute phase, also known as the “critical period” [32]. We applied dual tDCS synchronized with OT during this period, which is advantageous for maximizing brain plasticity and promoting upper limb function recovery to a greater extent. Secondly, as a general rule, the two hemispheres of the brain interact to balance excitation and inhibition. However, after a stroke, there is a reduction in excitability in the affected hemisphere, leading to compensatory hyperexcitability in the unaffected hemisphere, further reducing activity in the affected hemisphere and resulting in a severe imbalance between the two hemispheres, negatively impacting upper limb motor function [33]. Dual tDCS, with a weak current flowing between the anode and cathode, can modulate the resting membrane potential of underlying neurons in both M1 areas, altering cortical excitability [13]. It can induce long-term potentiation-like changes, promote functional reorganization and plasticity changes in the somatosensory-motor network, enhance motor learning, and improve upper limb function [34]. In addition, tDCS improves cerebral blood supply by mechanisms such as neurovascular coupling, increasing local cerebral blood flow, which can help reduce inflammation and protect ischemic areas’ neurons [35]. Finally, tDCS has a synergistic effect with OT. The synchronous combination of these two interventions involves central intervention and peripheral intervention. Central intervention works by promoting activation of functional brain areas, enhancing neuroplasticity, exciting motor descending pathways. Peripheral intervention involves sensory input stimulation transmitted to the central nervous system, reinforcing positive feedback and input of sensory and motor control patterns to the central system. This forms a “closed-loop” feedback pathway, acting on specific brain areas or functionally related brain regions in patients, promoting brain function reshaping and neural reorganization, leading to more pronounced and sustained improvements in brain performance [36,37] and facilitating upper limb functional recovery. Some evidence suggested that the combination of tDCS and peripheral interventions enhanced the effects of peripheral interventions themselves. For example, Li et al. [38] demonstrated that dual tDCS combined with sensory-motor training improved upper limb sensory and motor function, increased ADL capacity, and alleviated depression and anxiety in subacute stroke patients. Hsu et al. [39] found that dual tDCS during task-oriented training promoted motor recovery in subacute stroke patients. These findings are consistent with our study results.

The lack of significant interaction effect in ARAT may be attributed to the hand’s finer functionality, a larger brain region involved in hand control, and a greater number of corresponding neural fibers, making recovery slower and more challenging. The weak current provided by tDCS may have been insufficient to surpass the additional effects of OT, and the overall intervention duration may have been too short for tDCS effects to reach a significant level, resulting in a less pronounced tDCS effect on ARAT.

### EEG assessment

Quantitative EEG is a mature technology for assessing the functional state of the brain [40]. Stroke can lead to changes in brain rhythms in the resting state, typically characterized by a significant increase in delta activity and a decrease in alpha activity, resulting in high delta and low alpha activity [41,42]. This predominant slow-wave pattern is directly related to neural metabolism, reflecting ischemic and structural brain damage, and is associated with adverse outcomes after stroke [43]. DAR is the ratio of delta to alpha, integrating these spectral features and offering greater sensitivity than individual spectral components [44]. Finnigan et al. [45] collected EEG signals from 18 stroke patients and 28 healthy individuals, analyzing seven features of the EEG spectrum. The results showed that DAR had the highest accuracy in distinguishing stroke patients from healthy individuals, suggesting that DAR was an important indicator for assessing clinical treatment outcomes in stroke patients. In this study, over time, the M1-DAR significantly decreased in the experimental group, and between-group comparisons indicated that the experimental group outperforming the control group. Possible reasons for this are as follows. Firstly, during OT, the cerebral cortex can learn and store correct motor patterns through profound experiences, prompting the nervous system to establish correct motor cortex excitation traces. During tDCS, the stimulation can directly regulate the excitability of the motor cortex. In this study, we applied dual tDCS, with the anode electrode enhancing cortical excitability on the same side of M1 and the cathode electrode inhibiting cortical excitability on the opposite side of M1 [46], allowing for simultaneous modulation of excitability in both hemispheres of M1, thereby altering neural oscillatory power in bilateral M1 areas and reducing M1-DAR values. Secondly, tDCS can directly induce brain plasticity, increasing neuronal activity and gray matter volume in the motor cortex and pre-motor cortex [47,48], significantly enhancing interhemispheric functional connectivity and overall efficiency of the motor network [49], thereby altering neural oscillatory power and promoting normalization of brain electrical activity.

After treatment, while the global DAR decreased in all groups, there was no statistically significant difference compared to pre-treatment. This may be attributed to the limited range of tDCS effects and the relatively short intervention duration, preventing tDCS from rapidly exerting beneficial effects throughout the entire brain. However, it did have a strong effect on the targeted M1 area, promoting the normalization of activity in this brain region.

Stroke can also affect the activity of cortical areas involved by altering the spectral power distribution of the hemispheres. A power asymmetry between hemispheres can be represented by pdBSI [50]. Research by Sebastián-Romagosa et al. demonstrated the potential early predictive value of pdBSI for neurofunctional outcomes in stroke survivors, with higher values associated with poorer outcomes [51]. In this study, after treatment, only the experimental group showed a significant decrease in pdBSI, and between-group comparisons indicated that the experimental group outperformed the control group. This may be attributed to that dual tDCS can simultaneously act on both hemispheres, leading to bidirectional modulation of interhemispheric inhibition between the bilateral M1 areas, regulating cortical excitability effectively. This leads to a reduction in the normalized spectral density differences between the two hemispheres, thereby restoring the balance between the hemispheres, which is beneficial for enhancing neuroplasticity and improving upper limb function [52,53].

### Limitations

Despite a small number of patients in each group, the sample size was determined based on a power analysis. This study evaluated the outcomes at two time points within a two-week period and did not conduct long-term follow-up. Future studies could include larger sample sizes and incorporate long-term follow-up assessments. All patients included in this study had subcortical lesions, but specific lesion locations were not differentiated. In the future, it would be beneficial to categorize them into different subtypes based on the lesion locations to further investigate the effectiveness of treatment.

### Conclusion

This study provides evidence from both upper limb function and brain electrical activity perspectives, demonstrating the feasibility and effectiveness of the central-peripheral combined stimulation approach in subacute stroke patients. It indicates that real stimulation accelerates upper limb recovery, improves ADL abilities, and it also promotes the normalization of motor cortex EEG power and significantly improves the imbalance between the hemispheres compared to sham stimulation. However, further research is needed to investigate its additional effects on fine hand functionality. Importantly, these improvements were achieved within a relatively short training period (2 weeks). This suggests that the synchronous combination of dual tDCS and OT may be beneficial, offering a low-cost, effective, and relatively rapid protocol to upper limb motor recovery.

## Supporting information

S1 File CONSORT checklist. (DOC)

S2 File Trial protocol. (DOC)

S3 File Data used to build graphs. (XLSX)

S4 Table Table data. (DOC)
