# Supplementary material for: Effects of occupational therapy synchronized with dual transcranial direct current stimulation on upper limb function and electroencephalography power in subacute stroke patients: A randomized, double-blind, controlled study
Source: PLoS One. 2025 Mar 18;20(3):e0320142. doi: 10.1371/journal.pone.0320142 (PMC11918370; doi:10.1371/journal.pone.0320142)
Supplement: S2 File — (DOC) [file pone.0320142.s002.doc]

**Name of Research Project:** Effects of Occupational Therapy synchronized with Dual Transcranial Direct Current Stimulation on upper limb function and Electroencephalography Power in subacute stroke patients: a randomized, double-blind, controlled study

**Clinical Trial Protocol**

1. **Purpose of research**

The aim of this study is to explore the effects of dual transcranial direct current stimulation(dual-tDCS) synchronous occupational therapy(OT) on upper limb function and electroencephalogram(EEG) power in patients with subacute stroke, providing new treatment methods for upper limb rehabilitation after stroke.

**2. Inclusion and Exclusion Criteria**

**（1） inclusion criteria：**

(a) diagnosis with first unilateral subcortical stroke and has been confirmed by a head CT or MRI scan; (b) age between 25 and 80 years; (c) stroke with a stable clinical course lasting between 7 days and 6 months; (d) Brunnstrom staging of the upper limb and hand≤Stage IV; (e) Mini-Mental State Examination (MMSE) score≥21, indicating clear consciousness and ability to cooperate with the necessary examinations and treatment.

**（2）Exclusion criteria:**

(a) progressive stroke or subarachnoid hemorrhage with unstable condition; (b) patients with severe cardiovascular diseases, primary hypertension, internal medicine diseases, mental trauma, or cognitive impairments; (c) patients with metallic implants in the intracranial or treatment area, or with skull defects; (d) patients with epilepsy or brain tumors; (e) patients with poor compliance or unwillingness to cooperate with rehabilitation training.

1. **Specific Implementation Steps**

The authors confirm that all ongoing and related trials for this intervention are registered. This study was a randomized controlled trial, approved by the Ethics Committee of The Affiliated Xuzhou Rehabilitation Hospital of Xuzhou Medical University (NO.XK-LSW-20240321-018), and registered in the Chinese Clinical Trial Registry (www.chictr.org.cn) under the registration number ChiCTR2400082749. The individuals were recruited, evaluated, and completed treatment from The Affiliated Xuzhou Rehabilitation Hospital of Xuzhou Medical University and Xuzhou Central Hospital.

Group allocation was determined by a computer-generated random number sequence. The random numbers were placed in opaque sealed envelopes with corresponding numbers, which were kept by an investigator involved in participant recruitment. Patients were numbered in order of enrollment, and envelopes with matching numbers were opened to assign the corresponding treatment groups. All patients were allocated to either the control group or the experimental group in the same proportion. The control group received sham dual tDCS concurrent with OT, while the experimental group (n=22) received real dual tDCS concurrent with OT. The intervention was blind to participants, OT therapists, outcome assessor and statistician, with only the tDCS operator being unblinded. The specific method is as follows:

All the patients were given comprehensive OT by OT therapists who were blinded to the group assignments. Specific task activities were selected based on the patient's symptoms and specific conditions, including training for various joint movements of the upper limb (such as adduction and abduction of the shoulder joint, flexion and extension), upper limb muscle strength and endurance training (such as lifting a gymnastic rod and holding it), fine finger movement training (such as finger extension, gripping, opposition), and training for ADL (such as combing hair, buttoning clothes, eating), etc. Each session lasted for 40 minutes and was conducted once daily, five times a week, for a duration of two weeks. Additionally, the experimental group received real dual-tDCS at the same time while the control group received a sham stimulation. tDCS was applied using the A620P portable tDCS device (Nanjing Wogao Medical Technology Co., Ltd., China). The patients were comfortably seated with their whole body relaxed. The placement of tDCS electrodes was determined according to the international 10/20 system for electrode placement. Bipolar stimulation was applied, with the anode placed over the ipsilateral primary motor cortex (M1) area and the cathode placed over the contralateral M1 area [20]. The stimulation electrodes used standard configuration 5 cm × 5 cm saline-soaked sponge electrode pads, which were secured to the patient's head using elastic straps. The stimulation intensity was set at 2.0 mA, each session lasted for 20 minutes and was conducted once daily, five times a week, for a duration of two weeks. The same stimulation setup was used for sham tDCS, but the stimulation was stopped after 30 seconds.

1. **Judgment and Evaluation Methods of Experimental Research Efficacy**

All assessments will be performed by a clinically trained therapist who is blinded to the baseline data and group assignments at baseline and two weeks. Upper limb motor function and cortical EEG power were evaluated by Fugl-Meyer Assessment Upper Extremity (FMA-UE), Modified Barthel Index (MBI) and Action Research Arm Test (ARAT), Delta/Alpha Ratio (DAR) and pairwise derived Brain Symmetry Index (pdBSI) at baseline and two weeks.

4.1 Clinical evaluation

The primary outcome measure was Fugl-Meyer Assessment Upper Extremity (FMA-UE). FMA-UE is considered the gold standard for assessing motor impairment or motor control. It includes 33 items that evaluate reflexes, coordination, and isolated movements of the upper limb, with a total score of 66. A higher score indicates better upper limb function. The secondary outcome measures were Modified Barthel Index (MBI) and Action Research Arm Test (ARAT). MBI includes 10 items related to basic ADL, such as dressing, eating, and bathing. The total score is 100, and a higher score indicates better self-care ability for the patient. ARAT consists of 19 items that assess the ability to grasp, grip, pinch, and perform gross movements of the upper limb. The total score is 57, with a higher score indicating better upper limb function.

4.2 EEG data acquisition and analysis

The JY-2440 Digital Electroencephalography Topographic Mapping Instrument (Jiangsu Jinyuan Medical Technology Co., Ltd., China) was used to collect the patient's resting-state EEG data with closed eyes for a minimum of 5 minutes. Participants wore an EEG cap with 32 active electrodes positioned at FP1, FP2, F3, F4, C3, C4, P3, P4, O1, O2, F7, F8, T3, T4, T5, T6, AF3, AF4, FC3, FC4, CP3, CP4, PO3, PO4, FT7, FT8, CP7, CP8, FZ, CZ, PZ, and OZ, according to the international 10/20 system. Reference electrodes were placed at the bilateral earlobe locations, and a ground electrode was placed at the Z location. The EEG signals were filtered within the range of 0-30 Hz, with a time constant of 0.03 s and a sampling rate of 30 mm/s.

Data analysis was performed using MATLAB R2015a software and the EEGLAB toolbox. Independent Component Analysis (ICA) algorithm was used to correct artifacts. Fast Fourier Transform (FFT) was applied to convert the EEG signals into average power spectra for each electrode. The power spectra were then summed within the frequency bands of 1-4 Hz, 4-8 Hz, 8-12 Hz, and 13-30 Hz to obtain the average power values for delta, theta, alpha, and beta bands, respectively. These power spectra were used to calculate the following two quantitative indices.

1. Delta/Alpha Ratio (DAR): It is defined as the ratio between the absolute power of the delta band and the alpha band in EEG analysis.

DAR=
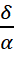


The "Average Scalp Power Spectra" was calculated by averaging the power across all scalp electrodes for each frequency iteration. The global DAR was then determined as the ratio between the average power of the delta band and the alpha band in the scalp power spectra.Similarly, the DAR in the primary motor cortex (M1) area (we refer to it as M1-DAR) was calculated using the average power from three electrodes: C3, C4, and CZ. Finally, at the group level, topographic maps of the global DAR and M1-DAR were generated to visualize the data, show the spatial distribution of these ratios across the scalp and provide detailed representations of DAR values across different electrode locations, aiding in the localization of specific active brain regions.

1. pairwise derived Brain Symmetry Index (pdBSI): It is defined as the average absolute normalized difference in spectral power between homologous channel pairs of the left and right hemispheres. It represents the average absolute value of the difference in power between the two hemispheres within the frequency range of 1-25 Hz, and reflects the symmetry between the two hemispheres of the brain.

pdBSI =
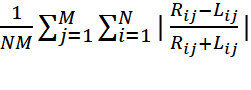


Here, R_ij and L_ij respectively represents the power of right and left channel from the homologous channel pair (for channel pair j = 1, 2, ..., M) at frequency i = 1, 2, ..., N. The pdBSI ranges from 0 to 1, where a pdBSI value of 0 indicates complete symmetry (total symmetry), and a value of 1 represents complete asymmetry (maximum asymmetry).

1. **Statistical Analysis**

The study protocol was based on a previous study published by Nair et al. That study investigated the effect of concurrent use of cathodal tDCS during OT on promoting the recovery of upper limb function in stroke patients. A total of 14 patients were recruited and randomly assigned to two groups, receiving real (cathodal) tDCS + OT and sham tDCS + OT respectively. The focus was on the changes in FMA-UE scores and joint range of motion. The results showed that the synchronous combination of cathodal tDCS and OT led to a significant improvement in post stroke motor function. The data of the above-mentioned study was used for power analysis. Taking the FMA-UE scores as the primary outcome, the sample size required for this study was calculated. The calculation was performed using PASS 15.0 software, assuming a power of 0.80 for a two-group comparison. The effect size (Cohen's d) for FMA-UE was estimated to be 0.47. Considering an estimated dropout rate of 20%, the conclusion was that 44 participants were required.

All analyses were performed by an independent assessor using SPSS 27.0 software. The normality of all continuous variables was assessed using the Shapiro-Wilk test. The homogeneity of variances was tested using the Levene's test. For baseline evaluations, Fisher's exact test was used to compare categorical data, while the independent samples t-test and Mann-Whitney U test were used to compare continuous data. For continuous variables that met the criteria of normal distribution and homogeneity of variances, two-way mixed analyses of variance (ANOVA) was performed. We chose to use it because it could allow for a more comprehensive and effective analysis of the changes between the experimental and control groups at different time points, enabling us to assess the impact of both factors and their interaction on the results. Time was used as the within subject factor (before and after treatment), and group was used as the between subject factor (control group and experimental group). In the case of a significant time × group interaction effect, Bonferroni adjustment was applied to the post-hoc pairwise comparisons of time and group to determine the comparisons that led to the differences. For continuous variables that did not meet the normality assumption, the Wilcoxon signed-rank test was used for within-group comparisons, and the Mann-Whitney U test was used for between-group comparisons. A p-value of < 0.05 was considered statistically significant.

**6. Technology Roadmap**

Assessed for eligibility

Enrolled

Assessment after treatment

(FMA-UE, MBI, ARAT, DAR and pdBSI)

Control group: sham dual-tDCS+OT

Experimental group: real dual-tDCS+OT

Assessment before treatment

(FMA-UE, MBI, ARAT, DAR and pdBSI)

Conduct statistical analysis and draw conclusions
