# Supplementary material for: Effects of occupational therapy synchronized with dual transcranial direct current stimulation on upper limb function and electroencephalography power in subacute stroke patients: A randomized, double-blind, controlled study
Source: PLoS One. 2025 Mar 18;20(3):e0320142. doi: 10.1371/journal.pone.0320142 (PMC11918370; doi:10.1371/journal.pone.0320142)
Supplement: S4 Table — (DOC) [file pone.0320142.s004.doc]

**Table 1. Baseline characteristics among the two groups.**

| Characteristic | Control group | Experimental group | p-value |
| --- | --- | --- | --- |
| **Age** **(years)** |  |  | 0.352 |
| Mean (SD) | 61.05(11.58) | 57.00(14.95) |  |
| **Sex** |  |  | 1.000 |
| Male | 13(68.42%) | 14(70.00%) |  |
| Female | 6(31.58%) | 6(30.00%) |  |
| **Time since stroke** **(days)** |  |  | 0.607 |
| Median (Q1, Q3) | 46(23.00, 60.00) | 40(24.50, 50.75) |  |
| **Impairment side** |  |  | 0.320 |
| Left hemiplegia | 14(73.68%) | 11(55.00%) |  |
| Right hemiplegia | 5(26.32%) | 9(45.00%) |  |
| **Stroke type** |  |  | 0.716 |
| Ischaemic | 15(78.95%) | 14(70.00%) |  |
| Haemorrhagic | 4(21.05%) | 6(30.00%) |  |

SD:standard deviation; Q1:1st quartile; Q3:3rd quartile.

Table 2. Comparison of clinical evaluation outcomes among the two groups.

| Variable/  Time | Control group | Experimental group | Time effect | Group effect | Time×Group effect | POST-HOC |
| --- | --- | --- | --- | --- | --- | --- |
|  | Mean ± SD | Mean ± SD |  |  |  | Exp vs Con |
| **FMA-UE** | | | | | | |
| Pre-treatment | 18.16±7.82 | 17.75±6.98 | F=195.402  p<0.001^*^  ղ^2^ =0.841 | F=5.567  p=0.024^*^  ղ^2^ =0.131 | F=42.326  p<0.001^*^  ղ^2^ =0.534 | p=0.864 |
| Post-treatment | 25.05±8.55 | 36.65±8.36 |  |  |  | p<0.001^*^ |
| **MBI** | | | | | | |
| Pre-treatment | 49.26±11.51 | 47.25±9.18 | F=315.682  p<0.001^*^  ղ^2^ =0.895 | F=0.854  p=0.361  ղ^2^ =0.023 | F=24.352  p<0.001^*^  ղ^2^ =0.397 | p=0.549 |
| Post-treatment | 62.63±12.22 | 70.90±11.17 |  |  |  | p=0.034^*^ |
| **ARAT** | | | | | | |
| Pre-treatment | 8.95±5.71 | 10.20±6.71 | F=210.974  p<0.001^*^  ղ^2^ =0.851 | F=1.190  p=0.282  ղ^2^ =0.031 | F=2.920  p=0.096  ղ^2^ =0.073 |  |
| Post-treatment | 17.63±8.13 | 21.20±8.02 |  |  |  |  |

SD: standard deviation.

*Significant effect (P < 0.05).

**Table 3. Comparison of EEG evaluation outcomes between the two groups.**

| Variable/  Time | Control group | Experimental group | Within-  Group (Con) | Within-  Group (Exp) | Between groups |
| --- | --- | --- | --- | --- | --- |
|  | Median  (Q1, Q3) | Median  (Q1, Q3) |  |  |  |
| **global DAR** | | | | | |
| Pre-treatment | 3.02  (2.04, 7.75) | 3.89  (2.20, 5.06) | p=0.136 | p=0.104 | p=0.857 |
| Post-treatment | 3.12  (1.68, 4.60) | 2.68  (1.36, 4.09) |  |  | p=0.708 |
| **M1-DAR** | | | | | |
| Pre-treatment | 3.12  (1.77, 6.07) | 3.63  (2.04, 5.41) | p=0.355 | p=0.019^*^ | p=0.667 |
| Post-treatment | 3.16  (1.84, 4.48) | 1.85  (0.86, 2.62) |  |  | p=0.022^*^ |
| **pdBSI** | | | | | |
| Pre-treatment | 0.28  (0.23, 0.40) | 0.30  (0.21, 0.46) | p=0.319 | p=0.006^*^ | p=0.665 |
| Post-treatment | 0.27  (0.20, 0.36) | 0.19  (0.12, 0.26) |  |  | p=0.025^*^ |

Q1:1st quartile; Q3: 3rd quartile.

*Significant effect (P < 0.05).
